# Toward function-oriented, neuroscience-based spine care in older adults: a structured narrative review and translational synthesis

**DOI:** 10.3389/fpain.2026.1674951

**Published:** 2026-06-04

**Authors:** Carl P. C. Chen, Areerat Suputtitada

**Affiliations:** 1 Department of Physical Medicine and Rehabilitation, Chang Gung Memorial Hospital at Linkou, College of Medicine, Chang Gung University, Taoyuan, Taiwan; 2 Department of Rehabilitation Medicine, Faculty of Medicine, Chulalongkorn University, Bangkok, Thailand; 3 Principles and Practice of Clinical Research (PPCR) Program, Harvard T.H. Chan School of Public Health, Harvard University, Boston, MA, United States

**Keywords:** chronic spinal pain, pain neuroscience education, central sensitization, function-oriented care, regenerative rehabilitation, ultrasound-guided mechanical needling with sterile water injection, neuromodulation, biopsychosocial rehabilitation

## Abstract

Chronic spinal pain remains the leading cause of disability worldwide, disproportionately affecting aging populations. Traditional pharmacological and surgical approaches offer limited long-term efficacy and carry significant risks. This structured narrative review examines recent evidence (2020–2025) on function-oriented, neuroscience-based approaches to spine care in older adults, with a focus on non-pharmacological and regenerative strategies such as Pain Neuroscience Education (PNE), structured exercise, psychological therapies, and manual therapy, which have been shown to improve pain and functional outcomes across multiple studies. Adjunctive modalities, including extracorporeal shockwave therapy (ESWT), high-intensity laser therapy (HILT), and thermotherapy, may provide additional benefits through modulation of neuroimmune and vascular pathways. Regenerative interventions, particularly ultrasound-guided mechanical needling with sterile water injection, have shown promising findings in observational studies for addressing fibrosis and calcification. Biologic injectables (e.g., PRP, HA, and MSCs) and neuromodulation techniques (rTMS and tDCS) also demonstrate potential but require protocol standardization and robust randomized trials. This review synthesizes contemporary evidence of varying methodological quality and proposes a tiered, function-oriented model that integrates these modalities into accessible, cost-effective, and globally relevant pain care systems.

## Introduction

Chronic pain, as a major public health challenge, defined as pain lasting longer than three months, affects more than 1.5 billion people globally and remains a leading cause of disability worldwide (1). Among chronic pain conditions, low back pain is responsible for the greatest number of years lived with disability (YLDs), particularly in aging populations and lower-resource settings (GBD 2021). The burden of chronic pain extends beyond physical symptoms, contributing significantly to mental health disorders, unemployment, and social exclusion.

Despite advances in research, chronic pain care continues to rely heavily on a biomedical model focused on structural abnormalities and nociceptive mechanisms (2–6). Pharmacological therapies, including opioids and corticosteroids, as well as invasive procedures, remain widely used, even as evidence suggests limited long-term effectiveness and potential adverse outcomes². Surgical interventions, while beneficial in selected cases, are often pursued despite uncertain structure–symptom relationships or poorly defined functional indications (3).

From a public health perspective, the economic burden of chronic pain is substantial. In the United States alone, the annual cost exceeds $600 billion, including both direct healthcare expenditures and productivity losses (7). Globally, under-treated or inadequately managed pain contributes to increased healthcare utilization, medication overuse, and long-term disability. These realities underscore the need for a shift from reactive, symptom-focused care toward proactive, function-oriented, and patient-centered models of management (4–6).

Although this call for change is not new, implementation remains inconsistent. Clinical guidelines from organizations such as the Centers for Disease Control and Prevention (CDC), the National Institute for Health and Care Excellence (NICE), and the World Health Organization (WHO) recommend non-pharmacologic interventions as first-line strategies for chronic pain management (4–6). However, uptake is often limited by systemic factors, including gaps in provider training, reimbursement constraints, and cultural expectations.

Importantly, these challenges are particularly relevant in older populations, where chronic spinal pain frequently coexists with frailty, multimorbidity, sarcopenia, and polypharmacy. These factors may influence both pain mechanisms and treatment responses, while also increasing the risk of falls, functional decline, and reduced adherence to rehabilitation.

Accordingly, effective pain management in aging populations requires approaches that are not only evidence-informed but also safe, accessible, and aligned with the complex clinical context of older adults. Within this evolving framework, psychological therapies, including cognitive behavioral therapy and acceptance-based approaches, are recognized as foundational components of interdisciplinary pain care, addressing maladaptive pain beliefs, emotional responses, and behavioral patterns that influence long-term outcomes. These interventions should therefore be integrated across all stages of management within a comprehensive biopsychosocial model.

This review is intended for clinicians working in multidisciplinary and interdisciplinary pain management settings, including physicians, rehabilitation specialists, and allied health professionals involved in the care of patients with chronic spinal pain.

## Objectives

This review aims to synthesize and critically interpret chronic pain management approaches in light of recent advances in neuroscience, rehabilitation science, pain physiology, and psychological research. It examines the shift from a symptom-focused paradigm toward approaches that prioritize functional restoration, autonomy preservation, and the extension of healthspan, particularly in aging populations disproportionately affected by chronic spinal pain.

Drawing on recent literature of varying methodological quality (2020–2025), this synthesis presents a clinically oriented and hypothesis-generating framework that integrates biological, psychological, and social determinants of pain. The proposed model reflects the multifactorial nature of chronic pain and is intended to support clinical reasoning and inform future research and policy development.

By bridging mechanistic understanding with real-world implementation considerations, this review seeks to contribute to evolving global health priorities and to support the development of sustainable, patient-centered, and context-sensitive care approaches across diverse healthcare systems.

## Methods

This study was conducted as a structured narrative review with critical synthesis of the literature. Peer-reviewed publications from January 2020 to March 2025 were identified through systematic searches of PubMed, Scopus, and Web of Science.

The search strategy combined controlled vocabulary (e.g., MeSH terms) and free-text keywords related to chronic spinal pain and its interdisciplinary management. Key terms included “chronic pain,” “low back pain,” “central sensitization,” “nociplastic pain,” “biopsychosocial model,” “exercise therapy,” “manual therapy,” “pain neuroscience education,” “extracorporeal shockwave therapy,” “high-intensity laser therapy,” “thermotherapy,” “mechanical needling,” “sterile water injection,” “ultrasound-guided sterile water injection,” “ultrasound-guided injection,” “ultrasound-guided needling,” “hydrodissection,” “fascial hydrodissection,” “interfascial hydrorelease,” “perineural injection,” “platelet-rich plasma,” “hyaluronic acid,” “mesenchymal stem cells,” “neuromodulation,” “transcranial direct current stimulation,” “repetitive transcranial magnetic stimulation,” as well as psychological and behavioral terms including “cognitive behavioral therapy,” “psychological therapy,” and “biopsychosocial intervention”.

Eligible studies included randomized controlled trials, systematic reviews, meta-analyses, and well-designed observational studies (e.g., cohort and case-control studies) relevant to integrative, function-oriented, and neuroscience-informed care. Studies were selected based on relevance to the research objectives, methodological rigor, and clinical applicability.

A formal systematic review or meta-analysis was not undertaken due to substantial heterogeneity in study designs, intervention protocols, outcome measures, and patient populations, which precluded meaningful quantitative pooling and direct comparison of effect sizes. Accordingly, a structured narrative approach was selected to enable integration of mechanistic, clinical, and translational evidence within a clinically meaningful framework.

Study selection was conducted through iterative screening of titles, abstracts, and full texts. Eligible studies were categorized into thematic domains, including non-pharmacological rehabilitation, regenerative interventions, neuromodulation, and psychological therapies. This structured approach enabled transparent organization and synthesis of heterogeneous evidence while maintaining clinical relevance.

Data were synthesized qualitatively, with emphasis on clinical applicability, consistency of findings, and translational relevance across care settings. Consistent with this approach, methodological limitations and overall strength of evidence were appraised narratively rather than through formal pooled risk-of-bias assessment. Although a formal PRISMA framework was not applied, the review followed structured principles of transparency and thematic synthesis to enhance methodological rigor.

## Evidence synthesis and critical interpretation

### The invisible burden: disconnect between imaging and pain experience

Despite advances in research, chronic pain care continues to rely heavily on a biomedical model focused on structural abnormalities and nociceptive mechanisms. However, this model may not fully capture the complexity of patients' lived experiences. One of its most critical shortcomings is the well-documented disconnect between structural imaging findings and clinical symptomatology. Degenerative spinal changes are frequently observed in asymptomatic individuals, raising questions about the clinical relevance of such radiological abnormalities.

Boden et al.'s seminal study revealed that a substantial proportion of asymptomatic subjects exhibited lumbar MRI abnormalities, such as disc herniation and facet arthropathy, despite reporting no symptoms (8). This observation has been further supported by Brinjikji et al., whose meta-analysis demonstrated that degenerative MRI findings (e.g., disc bulges and protrusions) are prevalent even among pain-free individuals, particularly with advancing age (9). More recent investigations have extended these findings across diverse populations. Smith et al. reported that 24.2% of healthy adults demonstrated asymptomatic cervical spinal cord compression, with increasing prevalence in older individuals (10). In another example, Bezuglov et al. found that over 90% of professional soccer players exhibited at least one degenerative lumbar spine condition on imaging despite being clinically asymptomatic, underscoring the limited specificity of structural findings in explaining pain, even in high-functioning populations (5).

This mismatch becomes even more consequential when individuals with minimal imaging abnormalities present with severe pain and functional impairment. Such discrepancies have prompted increased attention to central sensitization (CS), a neurophysiological process in which the central nervous system exhibits heightened sensitivity to both painful and non-painful stimuli. CS has been proposed as a potential explanatory mechanism for the persistence or amplification of pain in the absence of ongoing tissue damage, including cases of persistent pain following surgery or tissue healing (11, 12).

Neuroscientific advances have further supported this evolving perspective. Functional MRI studies have demonstrated alterations in brain networks, including changes in default mode network activity and descending inhibitory control, suggesting a role for maladaptive central processing in chronic pain. These findings have contributed to the recognition of nociplastic pain as a distinct pain category, characterized by altered nociception without clear evidence of tissue damage or somatosensory system disease (12–14).

Despite increasing recognition of the multidimensional nature of chronic pain, healthcare systems remain variably equipped to address its complexity. Many clinicians receive limited formal education in contemporary pain neuroscience, which may contribute to challenges in identifying and managing centrally mediated conditions such as nociplastic pain (13–16). In addition, diagnostic and reimbursement structures often continue to prioritize structural imaging and pharmacologic interventions, sometimes at the expense of functional assessments, psychosocial evaluation, and non-pharmacologic strategies, despite growing evidence supporting their role in chronic pain management (5, 6, 16).

This misalignment may contribute to suboptimal care pathways: individuals with incidental radiologic findings may undergo unnecessary interventions, while those with predominantly nociplastic or centrally mediated pain mechanisms may remain under-recognized or undertreated (13–17). The downstream consequences, including persistent pain, functional limitation, psychological distress, and reduced quality of life, represent an important and ongoing public health concern.

Addressing this gap requires a conceptual shift toward a multidimensional understanding of pain grounded in the biopsychosocial model. Priorities include strengthening education in pain neuroscience, enhancing diagnostic frameworks to better capture central mechanisms, and aligning healthcare systems with interdisciplinary, evidence-informed approaches. Importantly, moving beyond image-centric paradigms toward function-oriented strategies may support improvements in quality of life and long-term health outcomes, particularly in aging and high-risk populations.

### Advancing non-pharmacologic and non-surgical interventions

Recent high-level evidence provides a strong rationale for prioritizing non-pharmacologic strategies in chronic spinal pain. A Level I Bayesian network meta-analysis (18) demonstrated that active interventions, particularly structured exercise-based programs, consistently yield superior improvements in pain and functional outcomes compared with passive modalities alone. Combined approaches may offer incremental benefits in selected subgroups, particularly those with higher baseline disability or movement-related fear.

Within this framework, structured exercise therapy is widely regarded as a central component of management across chronic musculoskeletal conditions. Mechanistically, its effects extend beyond symptom reduction, encompassing proposed mechanisms such as systemic anti-inflammatory signaling, enhancement of proprioceptive input, activation of endogenous opioid pathways, and modulation of central pain processing. These multi-level adaptations are particularly relevant in nociplastic states characterized by central sensitization (17, 18).

Pain Neuroscience Education (PNE) and graded exposure to movement further extend the impact of exercise-based rehabilitation by targeting maladaptive pain cognitions and behaviors. By reframing pain as a neurophysiological process, PNE has been shown to reduce fear-avoidance, enhance self-efficacy, and facilitate engagement in active therapies, while graded exposure supports functional restoration and long-term behavioral adaptation (19). Importantly, psychological therapies are considered first-line, evidence-based components of chronic pain management and should be integrated alongside physical and educational interventions (17, 20).

Manual therapy may be considered a complementary modality within this framework. While techniques such as mobilization and myofascial release may provide short-term symptom relief through peripheral mechanisms, their primary role is to facilitate participation in active rehabilitation rather than to serve as stand-alone interventions (11, 20).

As healthcare systems increasingly prioritize conservative and patient-centered strategies for chronic pain management, adjunctive non-invasive modalities, including extracorporeal shockwave therapy (ESWT), high-intensity laser therapy (HILT), thermotherapy, and mechanical traction, are increasingly incorporated within multimodal rehabilitation frameworks. Emerging evidence suggests that these interventions may influence both peripheral nociceptive mechanisms and central pain processing, including pathways related to central sensitization and nociplastic pain (20, 21). A structured summary of mechanisms, advantages, limitations, and strength of evidence across these interventions is provided in Supplementary Table S1.

### Extracorporeal shockwave therapy (ESWT)

Extracorporeal Shockwave Therapy (ESWT) exerts multifaceted therapeutic effects through an interplay of biomechanical stimulation and biologically mediated responses that influence both tissue integrity and pain processing. The delivery of high-frequency acoustic impulses induces localized mechanical stress and cavitation phenomena, which increase cellular membrane permeability and activate mechanosensitive signaling pathways. These processes have been associated with enhanced expression of endothelial nitric oxide synthase (eNOS) and vascular endothelial growth factor (VEGF), contributing to neovascularization, improved microcirculation, and optimized tissue oxygenation, all of which are essential for soft tissue repair and regeneration (22–26).

In addition to its vascular and regenerative effects, ESWT has been associated with modulation of inflammatory activity. Evidence suggests downregulation of pro-inflammatory cytokines, including tumor necrosis factor-alpha (TNF-α) and interleukin-1 beta (IL-1β), alongside relative upregulation of anti-inflammatory mediators such as interleukin-10 (IL-10). These changes may help mitigate persistent low-grade inflammation commonly observed in chronic spinal disorders and contribute to restoration of tissue homeostasis (22–26).

From a neurobiological perspective, ESWT may influence nociceptive signaling through both peripheral and central mechanisms, potentially involving modulation of neuropeptide release and neural excitability. These effects align with contemporary models of chronic pain that emphasize neuroimmune and mechanobiological interactions, and may contribute to reductions in pain sensitivity and improvements in functional performance (22–26).

At the intracellular level, ESWT has been reported to engage key signaling cascades involved in tissue repair and regeneration. Activation of mitogen-activated protein kinase (MAPK) pathways, including extracellular signal-regulated kinases (ERK1/2), has been linked to cellular proliferation and gene transcription associated with tissue remodeling. In parallel, modulation of Wnt/β-catenin signaling has been implicated in mesenchymal stem cell activity, angiogenesis, and extracellular matrix turnover (22–26).

Collectively, these mechanisms position ESWT as a biologically active, non-invasive modality capable of enhancing microvascular function, supporting collagen synthesis, and modulating inflammatory and nociceptive processes. Its clinical utility is particularly evident in conditions characterized by impaired perfusion and reversible soft tissue dysfunction (26).

However, ESWT does not directly eliminate established fibrotic adhesions or dense calcific deposits. In clinical scenarios involving advanced structural pathology, such as calcific tendinopathy or facet-related degeneration, its effects may be limited when used in isolation (25–26). Emerging hydrodissection-based strategies using sterile water have also been explored as drug-sparing adjunctive interventions within multimodal rehabilitation frameworks, particularly for mechanically restricted pain conditions associated with calcification and fibrosis (26–28). These limitations support its integration within a multimodal treatment strategy, where complementary approaches may be required to address structural constraints alongside biological modulation.

### High-Intensity Laser Therapy (HILT)

High-Intensity Laser Therapy (HILT) is a non-invasive photobiomodulation modality that has demonstrated potential in the management of chronic musculoskeletal and spinal pain. Evidence from randomized controlled trials and systematic reviews indicates that HILT can reduce pain intensity and improve functional outcomes across a range of musculoskeletal conditions, with emerging applicability to spinal pain syndromes (29).

The therapeutic effects of HILT arise from a combination of photothermal and photobiological mechanisms. The photothermal component increases local tissue temperature, promoting vasodilation, enhanced blood flow, and muscle relaxation, which may alleviate paraspinal muscle hypertonicity and improve connective tissue extensibility. In parallel, photobiomodulation effects involve mitochondrial activation, particularly through cytochrome c oxidase, leading to increased adenosine triphosphate (ATP) production and improved cellular energy metabolism (26, 29–31).

At the molecular level, HILT has been associated with modulation of inflammatory signaling pathways, including reductions in pro-inflammatory cytokines such as tumor necrosis factor-alpha (TNF-α) and interleukin-1 beta (IL-1β), alongside increased expression of anti-inflammatory mediators such as interleukin-10 (IL-10). These effects may contribute to attenuation of inflammatory processes and support tissue recovery in chronic pain conditions (26, 29–31).

From a neurophysiological perspective, HILT may influence nociceptive processing by reducing peripheral nociceptor excitability and enhancing endogenous pain inhibitory mechanisms. These effects may increase pain thresholds and reduce hyperalgesia, particularly in patients with features of central sensitization or nociplastic pain (26, 29–31).

Collectively, HILT represents a biologically active, non-pharmacologic modality that integrates thermal, cellular, and neuromodulatory effects to support pain reduction and functional recovery. Its clinical role is best considered within a multimodal rehabilitation framework, particularly in patients with mixed nociceptive and centrally mediated pain mechanisms.

However, despite these beneficial effects, current evidence remains limited by heterogeneity in treatment parameters and a relative lack of high-quality, spine-specific randomized trials. Further research is warranted to establish standardized protocols and clarify its long-term effectiveness within integrated pain management strategies.

### Targeted thermotherapy for chronic pain: neurovascular and molecular pathways of heat and cryotherapy

Thermotherapy, encompassing both heat and cold modalities, offers effective non-pharmacologic strategies for managing musculoskeletal and spinal pain. These interventions exert their effects through complex neural, vascular, muscular, and biochemical pathways, influencing both peripheral and central pain mechanisms.

Heat therapy has been shown to alleviate low back pain (LBP) via multifactorial mechanisms that modulate nociceptive input and promote tissue healing. Localized heat application activates cutaneous thermoreceptors, which may inhibit pain transmission at the spinal level through gate control-like mechanisms, thereby reducing pain perception (32). Simultaneously, stimulation of transient receptor potential vanilloid 1 (TRPV1) channels has been associated with enhanced antinociceptive signaling across spinal and supraspinal circuits (32).

The rise in local tissue temperature facilitates vasodilation, improves blood flow, and accelerates cellular metabolism which are key processes that promote tissue repair and facilitate the clearance of inflammatory mediators. Notably, even a modest increase of 1 °C can elevate metabolic activity by 10%–15%, improving tissue oxygenation and nutrient delivery (32).

At the musculoskeletal level, heat therapy reduces muscle tone, relieves spasms, and enhances soft tissue extensibility, thereby interrupting the pain–spasm–pain cycle commonly observed in LBP. It also improves the mobility of the thoracolumbar fascia by reducing hyaluronan viscosity, potentially reversing scar-like adhesions and enhancing fascial glide (32). Beyond immediate analgesia, these effects may contribute to longer-term modulation of peripheral and central sensitization. Clinical evidence supports the efficacy of continuous low-level heat wrap therapy (CLHT), typically applied for 8 h daily, as a safe, drug-free, and cost-effective intervention for improving pain, stiffness, and functional outcomes in both acute and chronic non-specific LBP (32).

Cryotherapy exerts therapeutic effects through a multimodal cascade involving vascular, neuromuscular, and molecular mechanisms that collectively reduce inflammation, alleviate pain, and preserve joint integrity, particularly in osteoarthritis (OA). Exposure to cold triggers rapid vasoconstriction followed by reactive vasodilation (the “hunting reaction”), which may help reduce synovial fluid accumulation, edema, and tissue swelling (32). Concurrently, reduced nerve conduction velocity and diminished nociceptor excitability are associated with short-term analgesia and decreased joint stiffness (33, 34).

At the molecular level, cryotherapy has been reported to suppress pro-inflammatory cytokines such as IL-1β, IL-6, TNF-α, NF-κB, VEGF, and PGE2, while enhancing the expression of anti-inflammatory mediators including IL-10. It also may help mitigate oxidative stress by downregulating iNOS and MPO and increasing antioxidant defenses via enzymes such as glutathione and superoxide dismutase (SOD) (33, 34). These molecular adaptations may contribute to inhibition of cartilage degradation by suppressing matrix metalloproteinases (MMPs) and COX-2, and further promote anti-inflammatory responses through modulation of ICAM-1 and the IL-6/IL-17A axis (33, 34).

Clinical trials and meta-analyses have validated the efficacy of both localized and whole-body cryotherapy in improving pain, joint mobility, and inflammatory markers in early-stage OA. A 2025 meta-analysis of 11 randomized controlled trials (*n* = 274) demonstrated that whole-body cryotherapy significantly reduced serum IL-1β (SMD = −2.08 pg/mL, *P* < 0.05) and increased IL-10 (SMD = 0.78 pg/mL, *P* < 0.05), with particularly pronounced benefits in athletic and obese populations (34). Moderate cold exposure (11–15 °C) appears to optimize therapeutic outcomes, whereas deeper hypothermia (<10 °C) may increase the risk of tissue injury without added benefit (34).

Furthermore, cryotherapy may inhibit collagenase activity and enhance the mechanical resilience of connective tissues, supporting long-term joint stability. These findings underscore cryotherapy's role as a safe, accessible, and cost-effective adjunct, especially valuable during acute flares or post-exertional inflammation. Cryotherapy may be particularly beneficial in the post-intervention phase, where modulation of inflammatory and neurovascular responses may facilitate recovery of tissue perfusion and support functional outcomes (33, 34).

### Mechanical traction

Traction therapy, commonly used in the conservative management of cervical and lumbar radiculopathies, aims to alleviate nerve root compression through vertebral distraction. Mechanistically, it may enlarge the intervertebral foramen, reduce disc protrusion, decompress nerve roots, and modulate nociceptive input via proprioceptive receptor stimulation. Manual traction further targets soft tissue flexibility and joint mobility, while mechanical traction offers more consistent force delivery. Despite these theoretical benefits, the clinical evidence remains mixed.

Colombo et al. reported that traction added to standard care for cervical radiculopathy resulted in statistically significant but not clinically meaningful improvements, based on low-quality evidence (35). For lumbar radiculopathy, Vanti et al. found that supine mechanical traction may provide short-term benefits when combined with physiotherapy, although such effects were not consistently observed in higher-quality trials (36, 37). A randomized study by Studnicki et al. demonstrated moderate improvements in pain and neurodynamic outcomes following manual traction, though effect sizes remained modest (38). Overall, traction may provide short-term symptomatic relief in selected patients, particularly when used alongside other rehabilitation strategies.

Importantly, cervical traction should be applied with caution, particularly in older adults and individuals with underlying vascular vulnerability (e.g., atherosclerosis, connective tissue disorders, or suspected cervical artery pathology). Rare but clinically significant adverse events, including syncope and ischemic stroke, have been reported in association with cervical spine interventions. Current evidence suggests that such events are more likely related to the exacerbation of pre-existing cervical artery dissection (CAD) or the mobilization of thromboembolic material, rather than a direct causal effect on a healthy artery (35, 36).

From a clinical perspective, cervical mechanical forces may act as a precipitating factor in susceptible individuals. Therefore, careful patient selection, attention to warning symptoms (e.g., new-onset headache, dizziness, or neurological deficits), and appropriate risk assessment are recommended prior to application.

In contrast, lumbar traction is generally considered to have a more favorable safety profile, with serious adverse events being rare when applied appropriately (37, 38). Reported side effects are typically mild and transient, including discomfort, increased low back pain, or temporary exacerbation of radicular symptoms.

However, caution remains appropriate in specific populations, particularly in older adults with osteoporosis, vertebral instability, malignancy, or acute inflammatory conditions. Excessive traction force or inappropriate positioning may increase the risk of symptom aggravation or structural complications (39, 40).

Lumbar traction does not share the same neurovascular risk profile as cervical traction, although its clinical effectiveness remains variable. Current evidence suggests that it may be considered as an adjunctive option within a broader rehabilitation approach (37, 40).

In summary, traction therapy may have a role in selected patients; however, its benefits appear modest and context-dependent, supporting an individualized and cautious approach to its clinical use.

### Regenerative therapies in chronic spinal pain: mechanisms, evidence, and global relevance

Low back pain affects over 600 million people globally and remains a leading cause of disability. Its rising burden, driven by aging populations, increasing BMI, and sedentary lifestyles, underscores the urgent need for scalable, non-opioid, and cost-effective interventions that address underlying biomechanical and metabolic contributors beyond symptomatic relief. Within this context, regenerative medicine has introduced a spectrum of biologically oriented strategies, including mechanical needling with sterile water injection, platelet-rich plasma (PRP), dextrose prolotherapy, hyaluronic acid (HA), and mesenchymal stem cell (MSC) therapies.

Ultrasound-guided hydrodissection has been described using various injectates, including saline and dextrose-based solutions (41–42). Among these, sterile water has emerged as a drug-free alternative, with evolving evidence suggesting potential benefits in selected clinical contexts (26–28). Ultrasound-guided mechanical needling combined with sterile water injection represents a structurally targeted regenerative approach. Under ultrasound guidance, this technique enables precise targeting of pathological tissue planes, facilitating mechanical fragmentation of fibrotic adhesions and calcified deposits while restoring fascial gliding through hydrodissection (27, 28). In addition, hypotonic stimulation may contribute to modulation of nociceptive signaling and local inflammatory responses. These combined effects may enhance microvascular perfusion, reduce peripheral sensitization, and promote adaptive tissue remodeling within degenerative musculoskeletal environments (26–28).

As a minimally invasive and drug-sparing intervention, this approach may be particularly relevant in older adults and individuals with multimorbidity or polypharmacy considerations. Importantly, it can be positioned within an integrative regenerative framework, where adjunctive injectates such as PRP or dextrose may be selectively incorporated based on underlying pathology and therapeutic goals (41–47). Clinical observations from large-cohort studies have reported consistent improvements in pain, mobility, and functional outcomes (27, 28), although these findings require further confirmation in controlled trials.

Platelet-rich plasma (PRP) represents one of the most extensively studied orthobiologic injectates in spinal disorders. Its therapeutic effects are mediated through the release of growth factors, including PDGF, VEGF, and TGF-β, which promote angiogenesis, extracellular matrix synthesis, and cellular proliferation, while also modulating inflammatory pathways and nociceptive signaling (43–47). However, clinical outcomes remain heterogeneous due to variability in preparation protocols, including leukocyte content, platelet concentration, and activation methods, which influence the biological activity and inflammatory profile of PRP (43–47). Consequently, the lack of standardized protocols continues to limit reproducibility and widespread clinical adoption.

In parallel, HA and MSC therapies represent advanced biologic strategies aimed at biomolecular regeneration (48, 49). High-molecular-weight HA exerts anti-inflammatory and matrix-modulating effects through CD44 and RHAMM receptor pathways, contributing to suppression of pro-inflammatory cytokines and promotion of extracellular matrix synthesis within the nucleus pulposus. In addition, HA provides a viscoelastic scaffold that supports cellular retention, controlled growth factor activity, and mechanical load distribution in degenerative environments (48). MSC-based therapies further expand this paradigm through paracrine signaling and immunomodulatory effects, with emerging evidence suggesting potential roles in restoring disc hydration, extracellular matrix integrity, and structural support. However, substantial heterogeneity in cell sources, delivery strategies, and study design limits the strength and generalizability of current evidence, and high-quality randomized trials remain limited (49).

Collectively, these approaches reflect a shift toward mechanism-based, biologically oriented spine care. Within this evolving landscape, structurally targeted interventions such as ultrasound-guided mechanical needling combined with sterile water injection represent a pragmatic and potentially cost-efficient strategy. This technique enables simultaneous targeting of multiple pathological components, including fibrotic adhesions, calcific deposits, and altered fascial planes, within a single procedural session, offering a versatile approach that may reduce treatment burden and resource utilization.

As a drug-free intervention, it may further reduce pharmacological exposure while maintaining clinical applicability across diverse patient populations. Emerging evidence suggests favorable outcomes in pain reduction, functional improvement, and procedural tolerability (26, 28). In addition, the absence of exogenous biologic agents or synthetic compounds may support a favorable safety profile. Nevertheless, long-term outcomes, comparative effectiveness, and standardized protocols remain to be established through rigorously designed controlled studies.

### Neuromodulation for nociplastic pain: central mechanisms and public health integration

While regenerative interventions primarily target peripheral nociceptive drivers, neuromodulation addresses dysfunctional central pain processing, which is particularly relevant in nociplastic pain phenotypes such as chronic low back pain (CLBP). Non-invasive techniques, including repetitive transcranial magnetic stimulation (rTMS) and transcranial direct current stimulation (tDCS), aim to modulate cortical excitability, restore descending inhibitory control, and attenuate central sensitization.

When applied to the primary motor cortex (M1), rTMS engages thalamocortical and cortico-brainstem networks, thereby enhancing endogenous analgesic pathways and facilitating neuroplasticity. Evidence from meta-analytic data supports its role in chronic pain management, particularly when combined with task-specific rehabilitation to reinforce functional gains (50). This interaction between neuromodulation and motor training reflects a mechanism-based approach that integrates central and peripheral processes.

In contrast, tDCS modulates neuronal membrane potentials through polarity-specific currents, resulting in facilitation or inhibition of cortical excitability depending on electrode configuration. Although generally safe and well tolerated, its clinical efficacy in CLBP remains inconsistent. Systematic evidence suggests modest benefits following repeated sessions; however, findings are limited by small sample sizes and methodological heterogeneity (51). Experimental data further indicate that single-session stimulation may not produce immediate measurable changes in cortical excitability or neuromuscular activation, although associations between cortical excitability and trunk muscle activation have been observed, suggesting a potential link between central modulation and motor control (52).

Emerging evidence indicates that CLBP involves not only altered nociceptive processing but also maladaptive motor control and cortical reorganization. The lumbar multifidus muscle, a key stabilizer of the spine, often demonstrates impaired activation and delayed recruitment, which has been associated with altered cortical representation. Neurophysiological studies have shown disrupted cortical mapping and intermuscular coordination, supporting the concept that persistent pain is linked to dysfunction across sensorimotor networks (53). These findings provide a mechanistic rationale for neuromodulatory interventions targeting M1 to restore motor control and improve functional stability.

However, several methodological limitations should be considered. Many neuromodulation trials are characterized by small sample sizes, variability in stimulation parameters, inconsistent sham conditions, and short follow-up durations. In addition, the lack of standardized neuromuscular and neurophysiological outcome measures limits comparability across studies. Despite these challenges, both rTMS and tDCS demonstrate favorable safety profiles and feasibility in outpatient and rehabilitation settings.

From a translational and public health perspective, non-invasive neuromodulation represents a scalable adjunct to multidisciplinary pain management. Its relevance is particularly notable in nociplastic pain conditions, where conventional pharmacologic and surgical approaches often yield limited benefit. The combination of low systemic risk, portability, and a strong neuroscientific basis supports its integration into function-oriented rehabilitation models. Future progress will depend on high-quality pragmatic trials, standardized protocols, and implementation strategies that address barriers related to training, accessibility, and healthcare system integration. Within a broader shift toward mechanism-based and function-focused care, neuromodulation may serve as a key component in personalized, tiered treatment pathways.

### Psychological integration across the continuum of care: a core component of function-oriented spine management

**Figure 1:**
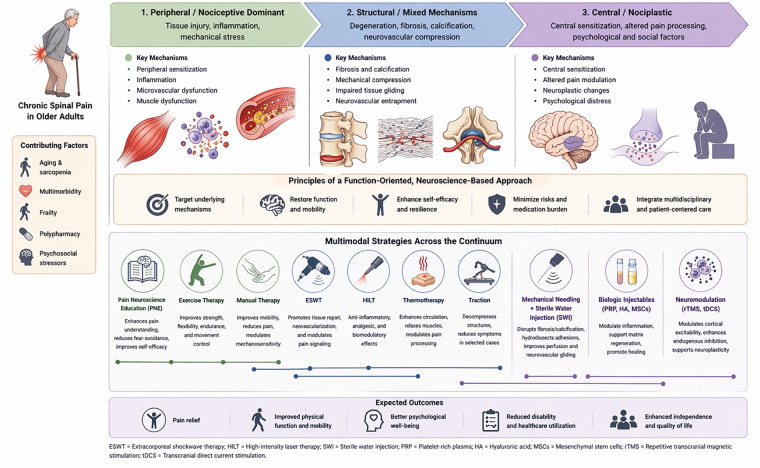
Mechanism-based continuum of chronic spinal pain in older adults. This figure depicts the continuum of chronic spinal pain across peripheral, structural, and central mechanisms, influenced by aging and comorbid factors. It highlights a function-oriented, neuroscience-based approach integrating multimodal, mechanism-targeted strategies to improve pain, function, and quality of life. Mechanical needling combined with sterile water injection primarily targets structural and peripheral pain mechanisms, while potentially exerting secondary modulatory effects on central pain processing through restoration of movement, reduction of nociceptive input, and functional recovery. Horizontal colored bars illustrate the relative overlap of interventions across pain mechanism domains.

**Figure 2:**
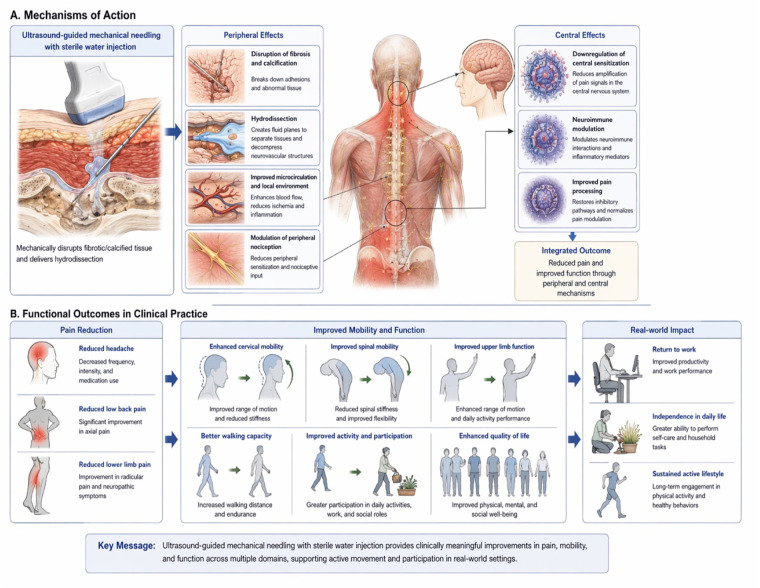
Functional outcomes following ultrasound-guided mechanical needling with sterile water injection. Improvements in pain reduction and mobility are observed in clinical practice, including reduced headache, enhanced cervical and spinal range of motion with decreased stiffness, improved walking capacity, and better upper limb function. These changes collectively support increased functional activity and participation in real-world settings.

**Figure 3:**
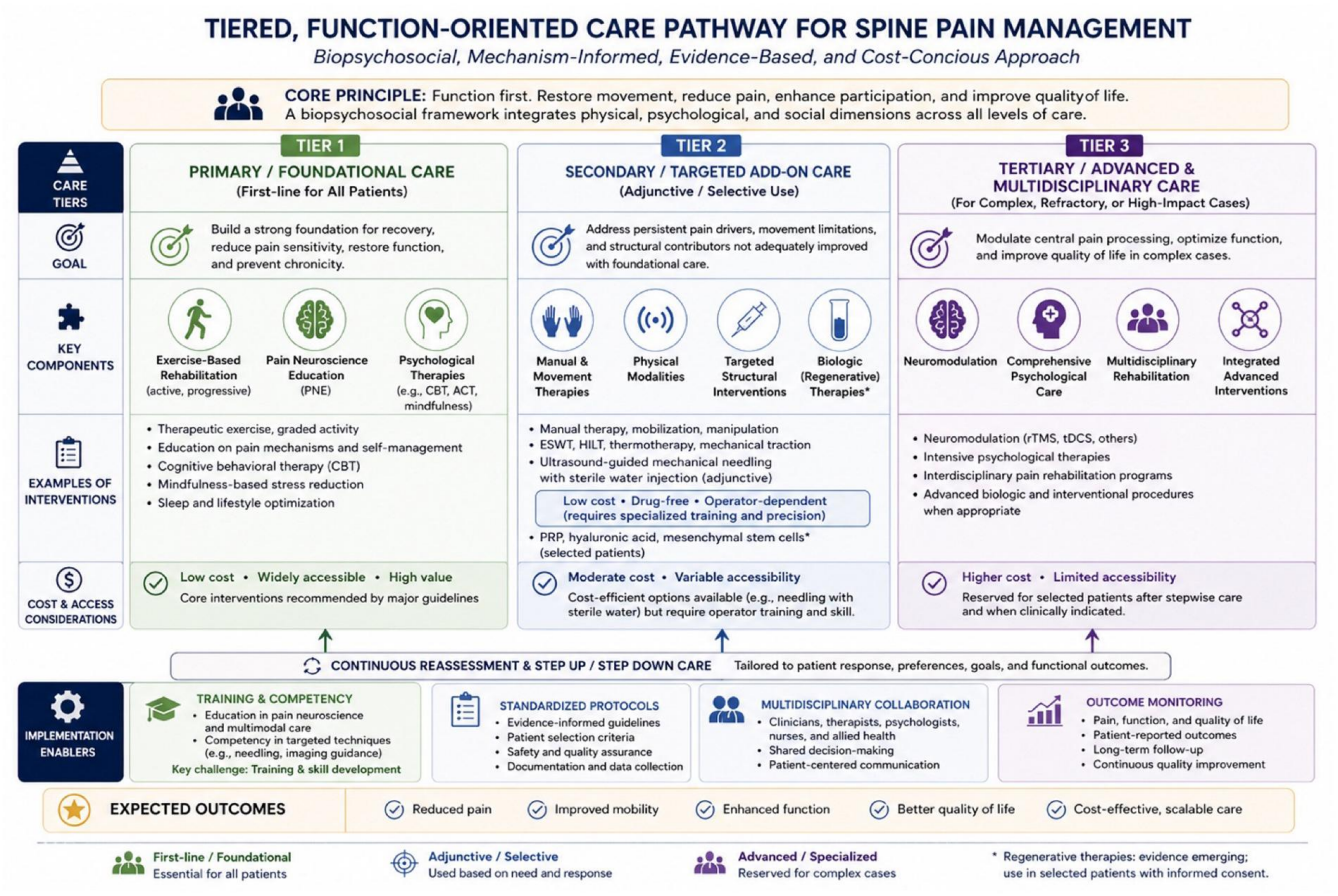
Tiered, function-oriented biopsychosocial care pathway for spine pain management. This figure illustrates a tiered, function-oriented care pathway for chronic spinal pain across primary (foundational), secondary (adjunctive), and tertiary (advanced) levels of care. Exercise-based rehabilitation, pain neuroscience education, and psychological therapies are positioned as first-line interventions across all stages. Secondary-level care includes targeted adjunctive approaches, such as manual therapy, physical modalities, and structurally directed interventions, including ultrasound-guided mechanical needling with sterile water injection. Tertiary care involves advanced and multidisciplinary strategies for complex or refractory cases. Short-term pharmacologic therapies may be used as supportive adjuncts during acute pain exacerbations to facilitate symptom control. The model emphasizes a biopsychosocial, evidence-informed approach, with consideration of cost, accessibility, and implementation in real-world clinical settings.

Beyond mechanism-based interventions, psychological factors remain integral across all stages of chronic spinal pain management. As illustrated in Figures 1–3, cognitive, emotional, and behavioral dimensions interact with peripheral, structural, and central pain mechanisms, influencing pain perception, treatment response, adherence, and long-term functional recovery. Evidence from a large Cochrane review indicates that cognitive behavioral therapy has the strongest evidence base among psychological therapies for chronic pain, with small but consistent benefits for pain, disability, and distress (54). Complementary evidence in chronic low back pain further supports the integration of psychosocial components with active physical interventions, demonstrating improved outcomes in pain intensity and disability compared with physical treatment alone (55). Accordingly, psychological considerations should accompany all therapeutic modalities throughout the continuum of care, ensuring that mechanism-informed spine care remains biopsychosocial, patient-centered, and function-oriented.

### Translating mechanism-based insights into function-oriented spine care

This review underscores the need to recalibrate chronic pain management through a neuroscience-informed, function-oriented, and integrative framework as illustrated in Figure 1. Traditional reliance on pharmacologic and surgical modalities may be insufficient to address key contributors to chronic pain, particularly those driven by central sensitization and nociplastic mechanisms. Although global health authorities recommend non-pharmacologic strategies as first-line approaches, implementation remains inconsistent, reflecting entrenched biomedical paradigms, limited training in pain neuroscience, and reimbursement structures that continue to prioritize procedural and pharmacological interventions (4–6).

Emerging evidence from 2020 to 2025 indicates that multimodal, non-invasive strategies are associated with improvements in pain, functional capacity, and patient-reported outcomes. Interventions such as structured exercise (17, 18), manual therapy (11, 20), and pain neuroscience education (19, 20) support functional restoration, psychological resilience, and patient self-efficacy. Adjunctive modalities, including ESWT (22–26), HILT (26, 29–31), and thermotherapy (32–34), mechanical traction (35–40) may provide additional benefit through integrated peripheral and central mechanisms, including vascular modulation, inflammatory regulation, and neuroimmune interactions.

Regenerative approaches are increasingly recognized as biologically plausible, patient-centered strategies. Among these, ultrasound-guided mechanical needling with sterile water injection has been associated with improvements in pain, mobility, and functional outcomes in large observational cohorts (27, 28). This technique enables structurally targeted intervention through mechanical disruption of fibrotic and calcified tissues, combined with hydrodissection to facilitate neurovascular decompression and restoration of fascial mobility.

Given its minimally invasive and drug-free nature, this approach appears to be well tolerated, with no consistent signals of serious adverse events reported to date. Accordingly, it may be considered for clinical application in appropriately selected patients, particularly in real-world settings where minimally invasive and drug-sparing strategies are prioritized.

However, procedural outcomes remain inherently operator-dependent, with effectiveness influenced by technical precision, anatomical targeting, and procedural expertise. Standardized training, competency-based frameworks, and protocol harmonization are therefore essential to ensure reproducibility and optimize clinical outcomes across diverse healthcare settings. In parallel, large-scale, longitudinal data collection at a global level will be critical to further define effectiveness, safety, and durability of outcomes, while supporting scalable and cost-efficient implementation across healthcare systems.

In clinical practice, improvements in walking capacity, low back pain, and lower limb pain have been consistently observed, with corresponding gains in functional mobility following intervention (27, 28). Importantly, as pain-related limitations often lead to reduced movement and activity, restoration of mobility and functional capacity represents a primary clinically meaningful outcome in real-world settings. These findings highlight the translational relevance of this approach in supporting active movement and daily function (26).

Additional functional benefits may extend beyond local pain reduction to include enhanced cervical mobility, reduced headache, and improved upper limb function. These observations are biologically plausible and may relate to improved neurovascular dynamics, including modulation of cervicogenic pathways and vertebrobasilar circulation. However, evidence supporting these extended effects remains limited and should be interpreted as hypothesis-generating, warranting confirmation in well-designed prospective studies (Figures 2, 3).

Biologic injectables, including PRP (43–47), HA (48), and MSCs (49), also demonstrate potential in modulating inflammation and supporting extracellular matrix regeneration. However, heterogeneity in formulation, dosing, and delivery techniques limits reproducibility and clinical translation. Accordingly, standardization of protocols and well-designed comparative studies are required to clarify their optimal clinical role and relative effectiveness.

Neuromodulation strategies, including rTMS and tDCS, target altered cortical processing and are particularly relevant in nociplastic pain phenotypes. rTMS has demonstrated potential in modulating descending inhibitory pathways and cortical networks (50), while tDCS may provide modest benefits depending on stimulation parameters and treatment intensity (51, 52). Nevertheless, variability in study design and protocol heterogeneity continue to limit the consistency and generalizability of reported outcomes.

Collectively, these findings support a paradigm shift from symptom suppression toward restoration of function and healthspan, emphasizing the integration of mechanism-informed, multimodal strategies within real-world clinical contexts.

Finally, psychological factors remain integral across all stages of chronic spinal pain management. As illustrated in Figure 3, this integrative framework demonstrates how cognitive, emotional, and behavioral dimensions interact with physical and structural mechanisms to influence pain perception, treatment response, and functional outcomes. Evidence from systematic reviews supports psychological therapies, particularly cognitive behavioral approaches, in improving pain, disability, and distress, reinforcing their role as a core component of multidisciplinary care (54, 55). Accordingly, these factors should be considered throughout the continuum of care, as they influence engagement, adherence, and long-term outcomes within a biopsychosocial, patient-centered framework.

From an implementation perspective, cost, accessibility, and scalability are central to intervention selection. In this context, ultrasound-guided mechanical needling with sterile water injection may offer a cost-efficient, drug-sparing approach, leveraging widely available ultrasound systems and a simple injectate (26–28).

Mechanistically, this approach enables precise targeting of structural pain generators, facilitating disruption of fibrotic adhesions and fragmentation of calcific deposits, with restoration of fascial gliding and segmental mobility. These effects may be accompanied by improved local perfusion, reduced neurovascular constraint, and modulation of peripheral nociceptive signaling, collectively supporting functional recovery (26–28).

In clinical practice, this technique is typically delivered over a limited number of sessions (often 1–4) and may allow treatment of multiple symptomatic regions within a single visit. This may reduce the need for repeated hospital attendance, lower indirect costs related to travel and time, and improve overall convenience for patients. Post-procedural care can often be streamlined, emphasizing early mobilization and self-directed exercise rather than resource-intensive, facility-based rehabilitation.

However, outcomes remain operator-dependent and require appropriate training, anatomical precision, and procedural expertise to ensure safety and reproducibility. These considerations are essential for scalable implementation across diverse healthcare settings.

From a translational and health systems perspective, cost, accessibility, and scalability are critical determinants of intervention selection. While advanced modalities such as extracorporeal shockwave therapy (ESWT) (22–26), high-intensity laser therapy (HILT) (26, 29–31), regenerative interventions (26–28, 41–49), and neuromodulation (50–52) demonstrate biological plausibility and emerging clinical potential, their availability, equipment requirements, and associated costs may limit widespread implementation, particularly in low- and middle-income settings. Accordingly, these interventions are best considered as adjunctive components within a broader, multimodal framework that prioritizes accessible, first-line strategies, including exercise-based rehabilitation (18), pain neuroscience education (19, 20), and psychological therapies (54, 55).

In this context, structurally targeted, ultrasound-guided mechanical interventions using sterile water may offer a pragmatic and resource-efficient option, supporting functional recovery while minimizing pharmacologic exposure (26–28). Importantly, this approach may also represent a cost-efficient and scalable solution in real-world settings. It is typically delivered over a limited number of sessions (often 1–4) and may allow treatment of multiple symptomatic regions within a single visit, thereby reducing healthcare utilization, minimizing the need for repeated hospital attendance, and lowering the overall patient burden.

### Mechanism-informed clinical pathways for function-oriented spine care

Chronic spinal pain in older adults can be approached through a mechanism-informed, function-oriented continuum of care that aligns therapeutic strategies with underlying pain biology while maintaining adaptability across diverse clinical contexts (Figure 1). This framework is further translated into a tiered, function-oriented biopsychosocial model of care, as illustrated in Figure 3, which prioritizes accessible, first-line interventions while integrating adjunctive and advanced strategies according to clinical complexity and resource availability.

Exercise-based rehabilitation, pain neuroscience education, and psychological therapies are positioned as first-line interventions across all stages of care (18–20, 53–55). Short-term pharmacologic therapies may be used as supportive adjuncts during acute pain exacerbations to facilitate symptom control (2, 4, 6).

#### Primary level: foundational care

Exercise-based rehabilitation and pain neuroscience education form the foundation of care, supporting functional restoration, modulation of pain perception, and patient self-efficacy (17–19, 53). Psychological therapies are integrated across all stages to address maladaptive pain-related beliefs, enhance coping strategies, and support long-term behavioral adaptation (54, 55).

#### Secondary level: adjunctive interventions

Adjunctive strategies may be introduced based on symptom persistence and clinical needs. These include manual therapy and physical modalities, such as extracorporeal shockwave therapy (ESWT) and high-intensity laser therapy (HILT), which have been associated with modulation of peripheral sensitization through angiogenic, anti-inflammatory, and tissue remodeling effects (22–26, 29–31). Thermotherapy may further enhance local circulation and neuromodulatory processes (32–34), while traction may provide short-term symptomatic relief in selected patients (35–40).

Structurally directed interventions may also be considered at this level. Ultrasound-guided mechanical needling with sterile water injection represents a drug-free, structurally targeted approach that may disrupt fibrosis, fragment calcifications, and facilitate hydrodissection. These mechanisms may improve local perfusion, reduce neurovascular entrapment, and restore fascial mobility within degenerative tissue environments (26–28), as illustrated in Figure 2.

In clinical practice, this approach may allow treatment of multiple symptomatic regions within a single visit, depending on clinical presentation and anatomical considerations. This may reduce the need for repeated hospital attendance, lower indirect costs, and improve overall patient convenience, thereby supporting real-world feasibility and scalability across healthcare settings.

In selected patients with structurally driven pain, targeted mechanical approaches may directly address key pathological substrates, including fibrosis and calcific deposition. By restoring tissue mobility and relieving mechanical constraints, such interventions may, in some cases, reduce the need for additional biologic or injectable therapies. However, treatment selection should remain individualized, and adjunctive modalities, including platelet-rich plasma (PRP) (43–47), hyaluronic acid (HA) (48), and mesenchymal stem cells (MSCs) (49), may still be appropriate depending on clinical context and patient-specific factors, acknowledging the heterogeneity of current evidence.

#### Tertiary level: advanced and multidisciplinary care

Advanced and multidisciplinary strategies may be considered for complex or refractory cases. These may include neuromodulation approaches, such as repetitive transcranial magnetic stimulation (rTMS) and transcranial direct current stimulation (tDCS), which may modulate cortical excitability and facilitate adaptive neuroplastic reorganization (50–53).

This model emphasizes a biopsychosocial, evidence-informed approach, with consideration of cost, accessibility, and implementation in real-world clinical settings, enabling personalized, scalable, and context-sensitive care.

## Limitations

This review has several limitations inherent to its structured narrative synthesis design. A formal systematic review or meta-analysis was not undertaken due to substantial heterogeneity in study designs, intervention protocols, outcome measures, and patient populations across the included literature. This variability precludes meaningful quantitative pooling and limits direct comparison of effect sizes across interventions. Accordingly, a structured narrative approach was adopted to integrate mechanistic, clinical, and translational evidence within a clinically relevant framework.

The absence of quantitative meta-analytic synthesis restricts formal estimation of treatment effects and reduces the ability to draw definitive comparative conclusions. In addition, heterogeneity in study populations, intervention delivery, and outcome definitions may affect both internal consistency and external validity of the synthesized findings.

Blinding of operators and participants is inherently challenging in many interventional and rehabilitation-based studies, including injection procedures and physical therapy interventions, due to the hands-on nature of treatment delivery. This limitation introduces potential performance and detection bias, particularly in studies relying on subjective outcomes such as pain and function.

Variability in intervention protocols, including differences in dosing, technique, and delivery parameters, further limits reproducibility and complicates interpretation across studies. In several domains, particularly regenerative and neuromodulatory interventions, the available evidence is also constrained by small sample sizes, short follow-up durations, and inconsistent reporting standards.

The restriction to contemporary literature (2020–2025) enhances relevance to current clinical practice and reflects recent advances in neuroscience-informed and function-oriented care. However, this approach may underrepresent earlier foundational mechanistic evidence that continues to inform current therapeutic paradigms.

Potential publication bias and selective outcome reporting remain important considerations, particularly in rapidly evolving fields where positive findings may be preferentially reported.

Finally, the predominance of evidence derived from high-income healthcare settings may limit generalizability to other contexts, particularly in low- and middle-income settings with differing healthcare infrastructure and resource availability.

## Future directions

Future research should prioritize harmonization of outcome measures and standardization of intervention protocols, including dosing, procedural techniques, and reporting frameworks, to enhance reproducibility and enable meaningful cross-study comparisons. Where feasible, well-designed randomized controlled trials and advanced quantitative synthesis are needed to strengthen comparative effectiveness evidence.

In addition to randomized controlled trials, alternative study designs should be considered to better capture the complexity of chronic spinal pain and real-world clinical practice. Pragmatic trials, adaptive trial designs, and hybrid effectiveness–implementation studies may offer greater external validity while maintaining methodological rigor.

Prospective cohort studies and registry-based research can provide valuable longitudinal data on safety, durability of treatment effects, and functional outcomes across diverse patient populations. In parallel, mixed-methods research incorporating patient-reported experiences may further enhance understanding of adherence, acceptability, and real-world impact of interventions.

Given the inherent challenges of blinding in interventional and rehabilitation-based studies, large-scale observational and real-world studies may serve as important complementary sources of evidence, particularly for evaluating safety, feasibility, and long-term functional outcomes in routine clinical practice. Such approaches are particularly relevant in aging populations and complex multimorbidity contexts, where conventional trial designs may not fully reflect real-world clinical decision-making.

Greater emphasis should also be placed on integrative research designs that capture the multidimensional nature of chronic spinal pain, incorporating mechanistic, functional, and patient-centered outcomes. Approaches such as comparative effectiveness research and pragmatic trials may help bridge the gap between controlled research environments and real-world clinical settings.

In parallel, future investigations should expand to diverse healthcare contexts, with particular attention to feasibility, cost-effectiveness, and equity in low- and middle-income countries. Embedding implementation science frameworks will be essential to facilitate translation of evidence into practice, including clinician training, patient education, and health system integration.

Ultimately, advancing chronic pain care will require coordinated efforts across clinical research, health policy, and education to support scalable, context-sensitive, and function-oriented models of care aligned with both biological mechanisms and lived patient experience.

## Conclusion

A mechanism-informed approach to chronic spinal pain can be conceptualized as a dynamic continuum reflecting the shifting dominance of pain mechanisms alongside evolving functional needs. In early stages, peripheral sensitization may predominate and respond to non-invasive interventions. With increasing structural contribution or persistence of symptoms, tissue-directed and regenerative strategies may become relevant. In more advanced or chronic stages, central sensitization and functional impairment often dominate, necessitating neuromodulatory and function-oriented rehabilitation approaches.

This continuum is not intended as a prescriptive pathway, but rather as an integrative conceptual framework to support clinical reasoning, individualized care, and context-sensitive decision-making across the spectrum of chronic spinal pain.

Overall, this review underscores a paradigm shift from symptom-centered management toward mechanism-oriented, function-centered care. The integration of non-pharmacologic, regenerative, and neuromodulatory strategies within a biopsychosocial framework provides a more comprehensive and sustainable approach to chronic pain management. Such an approach aligns with contemporary efforts to reduce reliance on pharmacologic and invasive interventions while prioritizing functional recovery, patient autonomy, and long-term health outcomes.

By bridging mechanistic understanding with clinically actionable strategies, this framework offers a scalable and evidence-informed pathway to improve healthspan and quality of life, particularly in aging and complex patient populations.

## Data Availability

The original contributions presented in the study are included in the article/Supplementary Material, further inquiries can be directed to the corresponding author.
