# Supplementary material for: Toward function-oriented, neuroscience-based spine care in older adults: a structured narrative review and translational synthesis
Source: Front Pain Res (Lausanne). 2026 Jun 4;7:1674951. doi: 10.3389/fpain.2026.1674951 (PMC13275645; doi:10.3389/fpain.2026.1674951)
Supplement: Supplementary file 1 [file Table1.docx]

**Supplementary Table 1. Mechanism-Based Comparison of Interventions in Chronic Spinal Pain: Evidence Levels and Study Characteristics**

| **Intervention** | **Mechanistic Rationale** | **Key Advantages** | **Limitations** | **Study Type / Sample Size** | **Strength of Evidence** | **Clinical Role** |
| --- | --- | --- | --- | --- | --- | --- |
| Structured Exercise Therapy^17,18^ | Central modulation, motor control restoration, anti-inflammatory and endogenous opioid activation | Improves pain and function, scalable, first-line | Requires adherence, heterogeneous protocols | Multiple RCTs, meta-analyses (large pooled samples) | High | Core treatment across all stages, especially nociplastic pain |
| Pain Neuroscience Education (PNE)^19,20^ | Cognitive modulation, reduces fear-avoidance, improves cortical processing | Enhances engagement and self-efficacy | Dependent on patient engagement | RCTs, systematic reviews | Moderate–High | Essential adjunct to support behavioral change |
| \| **Psychological Therapy (e.g., CBT)^54,55^** \| \| --- \| | \| Cognitive and affective modulation; reduces catastrophizing and fear-avoidance \| \| --- \| | \| Improves coping, adherence, and long-term outcomes \| \| --- \| | \| Requires trained providers; patient engagement \| \| --- \| | \| RCTs, systematic reviews \| \| --- \| | \| High \| \| --- \| | \| Core component across all stages \| \| --- \| |
| Manual Therapy ^11,20^ | Peripheral mechanoreceptor stimulation, short-term nociceptive modulation | Rapid symptom relief, improves mobility | Short-term effect, not standalone | RCTs, systematic reviews | Moderate | Adjunct to facilitate active rehabilitation |
| ESWT ^22-25^ | Mechanotransduction, angiogenesis (VEGF), and anti-inflammatory signaling | Non-invasive, promotes tissue repair and microcirculation | Limited effect in advanced fibrosis/calcification | RCTs, meta-analyses | Moderate–High | Early–mid stage (peripheral sensitization) |
| HILT ^29-31^ | Photobiomodulation, mitochondrial activation, anti-inflammatory pathways | Improves pain and functional outcomes | Protocol heterogeneity | RCTs, systematic reviews | Moderate | Adjunct for mixed pain mechanisms |
| Thermotherapy ^32-34^ | TRPV1 activation, vascular modulation, inflammatory control | Safe, accessible, low-cost | Mainly symptomatic relief | RCTs, meta-analyses | Moderate | Early-stage or symptom flare management |
| Mechanical Traction ^35-40^ | Reduces nerve root compression, modulates nociception | May relieve radicular pain short-term | Modest effect, inconsistent findings | RCTs, meta-analyses | Low–Moderate | Selected radiculopathy cases |
| Mechanical Needling + Sterile Water Injection ^27-28^ | Mechanical disruption of fibrosis/calcification, hydrodissection, mechanosensitive channel activation | Structurally targeted, drug-free, restores fascial mobility | Operator-dependent, limited RCTs | Large observational cohorts (>4,000 patients), consistent outcomes | Moderate–High | Mid-stage (structural pathology, calcification, fibrosis, entrapment) |
| PRP / Biologic Injectables ^41-49^ | Growth factor-mediated regeneration, ECM remodeling | Potential regenerative effects | High heterogeneity, lack of standardization | RCTs, systematic reviews (variable size) | Moderate | Selected degenerative conditions |
| Neuromodulation (rTMS, tDCS) ^50-52^ | Cortical modulation, enhances descending inhibitory pathways | Targets central sensitization | Small sample sizes, protocol variability | RCTs, meta-analyses (small–moderate samples) | Moderate | Late-stage / nociplastic pain |

Abbreviations: ESWT, extracorporeal shockwave therapy; HILT, high-intensity laser therapy; PRP, platelet-rich plasma; rTMS, repetitive transcranial magnetic stimulation; tDCS, transcranial direct current stimulation.

Strength of evidence reflects the overall consistency and quality of available studies, including randomized controlled trials, systematic reviews, and large observational cohorts.

Reference numbers correspond to the main manuscript reference list.
